# “YOU HAVE TO USE YOUR IMAGINATION”: NURSING HOME PROVIDER MODIFICATIONS TO IMPLEMENTING THE IPPI

**DOI:** 10.1093/geroni/igae098.0160

**Published:** 2024-12-31

**Authors:** Miranda Kunkel, Nahida Akter, Kamryn Kasler, Cassie Keiser, Molly Noble, Alexis Talmage, Kimberly Van Haitsma, Katherine Abbott

**Affiliations:** Collaborative Consulting, Oxford, Ohio, United States; The Pennsylvania State University, University Park, Pennsylvania, United States; Miami University, Oxford, Ohio, United States; Miami University, Oxford, Ohio, United States; Miami University, Oxford, Ohio, United States; Miami University, Oxford, Ohio, United States; The Pennsylvania State University, University Park, Pennsylvania, United States; Miami University’s, Oxford, Ohio, United States

## Abstract

Understanding modification motive can help improve intervention protocols and future implementation efforts. The core components of the IPPI that should not be modified include that staff approach one-to-one resident interactions with a positive intent, use good communication skills, and base interactions on a preferred activity of the resident living with dementia. We sought to understand what was modified and motivations behind the modifications from implementation champions. We utilized the Framework for Reporting Adaptations and Modifications to Evidence-Based Implementation Strategies (FRAME-IS) to track and characterize modifications using data from a total of n=78 interviews with n=23 NH provider champions who were implementing the IPPI in their community as part of a quality improvement project. Interviews were audio recorded, transcribed, and coded via the FRAME-IS in Dedoose. Over half of champions (61%; n=14) reported unplanned, fidelity-consistent modifications to IPPI content (e.g., loosening structure) or implementation context (e.g., setting). Motivation ranged from organizational (e.g., time constraints) to recipient (e.g., cognitive capacity), with many citing resident preferences. Modifications are a necessary aspect of evidence-based implementation efforts and should be embraced by implementation researchers. Many champions in the present study reported creative modifications previously unexplored by the IPPI research team. Available resources, COVID-19 regulations, and resident preferences can highly impact implementation efforts in the NH setting. Future implementation efforts should build flexibility into intervention protocols and welcome the influence of recipient preferences in order to reach a wider range of users and achieve greater satisfaction.

